# Environmental and Occupational Interventions for Primary Prevention of Cancer: A Cross-Sectorial Policy Framework

**DOI:** 10.1289/ehp.1205897

**Published:** 2013-02-05

**Authors:** Carolina Espina, Miquel Porta, Joachim Schüz, Ildefonso Hernández Aguado, Robert V. Percival, Carlos Dora, Terry Slevin, Julietta Rodriguez Guzman, Tim Meredith, Philip J. Landrigan, Maria Neira

**Affiliations:** 1 Department of Public Health and Environment, World Health Organization (WHO), Geneva, Switzerland; 2 Section of Environment and Radiation, International Agency for Research on Cancer (IARC), Lyon, France; 3 Hospital del Mar Institute of Medical Research (IMIM–Hospital del Mar–prbb), Barcelona, Spain; 4 Networking Centre of Biomedical Research in Epidemiology and Public Health (CIBERESP), Spain; 5 Universidad Autónoma de Barcelona (UAB), Barcelona, Spain; 6 Department of Public Health, Facultad de Medicina, Universidad Miguel Hernández, San Juan de Alicante, Spain; 7 Environmental Law Program, University of Maryland Carey School of Law, Baltimore, Maryland, USA; 8 Cancer Council Western Australia, Perth, Western Australia, Australia; 9 Occupational Health Program, El Bosque University, Bogota, Colombia; 10 Department of Sustainable Development and Environment, Pan-American Health Organization/ WHO, Washington DC, USA; 11 Department of Preventive Medicine, Mount Sinai School of Medicine, New York, New York, USA

**Keywords:** cancer, environmental health, occupational, policy, primary prevention, public health

## Abstract

Background: Nearly 13 million new cancer cases and 7.6 million cancer deaths occur worldwide each year; 63% of cancer deaths occur in low- and middle-income countries. A substantial proportion of all cancers are attributable to carcinogenic exposures in the environment and the workplace.

Objective: We aimed to develop an evidence-based global vision and strategy for the primary prevention of environmental and occupational cancer.

Methods: We identified relevant studies through PubMed by using combinations of the search terms “environmental,” “occupational,” “exposure,” “cancer,” “primary prevention,” and “interventions.” To supplement the literature review, we convened an international conference titled “Environmental and Occupational Determinants of Cancer: Interventions for Primary Prevention” under the auspices of the World Health Organization, in Asturias, Spain, on 17–18 March 2011.

Discussion: Many cancers of environmental and occupational origin could be prevented. Prevention is most effectively achieved through primary prevention policies that reduce or eliminate involuntary exposures to proven and probable carcinogens. Such strategies can be implemented in a straightforward and cost-effective way based on current knowledge, and they have the added benefit of synergistically reducing risks for other noncommunicable diseases by reducing exposures to shared risk factors.

Conclusions: Opportunities exist to revitalize comprehensive global cancer control policies by incorporating primary interventions against environmental and occupational carcinogens.

Cancer is the second leading cause of death worldwide. In 2008, there were 7.6 million deaths from cancer, and 12.7 million new cancer cases (Ferlay et al. 2010). More than half of all cancers and 63% of cancer deaths occur in low- and middle-income countries.

Estimations show that at least one–third of all cancer cases could be prevented based on current knowledge (Danaei et al. 2005). Although preventable risk factors such as tobacco use, alcohol consumption, unhealthy diet, and physical inactivity play a major role in the development of cancer, a range of environmental factors and occupational exposures also contribute significantly to the global cancer burden (Parkin et al. 2011; President’s Cancer Panel 2010; Tomatis et al. 1990). Exposures to environmental and occupational carcinogens are often preventable.

“Environment” is defined by the World Health Organization (WHO) for the purpose of environmental attribution as “all the physical, chemical and biological factors external to the human host, and all related behaviors, but excluding those natural environments that cannot reasonably be modified” (Prüss-Ustün and Corvalán 2006). This definition is limited to those parts of the environment that can, in principle, be modified so as to reduce the impact of the environment on health. It also excludes those behaviors and lifestyles not strictly related to environmental exposures such as alcohol consumption and tobacco use as well as behaviors related to the social and cultural environment, genetics, and parts of the “unmodifiable” natural environment (Prüss-Ustün and Corvalán 2006).

Humans are exposed to numerous carcinogenic agents through inhalation, eating, drinking, and skin contact. Since most people work for nearly two–thirds of their lifetime, they have many, and often prolonged, opportunities for contacts with occupational carcinogens, resulting in the accumulation of exposure over a lifetime. WHO has estimated that a substantial proportion of all cancers are attributable to the environment, including work settings (WHO 2009a). For 2004, it was estimated that occupational lung carcinogens (such as arsenic, asbestos, beryllium, cadmium, and chromium) caused 111,000 lung-cancer deaths, and asbestos alone was estimated to cause 59,000 deaths from mesothelioma. Moreover, it was estimated that outdoor air pollution caused 108,000 lung-cancer deaths globally (WHO 2009a). Environmental factors that increase risks for developing cancer typically affect the general population through involuntary exposures, over which individuals have little control. Exposure to most carcinogens tends to be greatest in the most disadvantaged segments of the population [International Agency for Research on Cancer (IARC) 1997].

Exposures to environmental and occupational carcinogens can be reduced or eliminated, and the cancers that result from them can be prevented through policies promoting healthy working and living environments (Prüss-Ustün and Corvalán 2006; Prüss-Ustün et al. 2011). Primary prevention encompasses the reduction or elimination of exposure to established risk factors to prevent the occurrence of disease (Tomatis et al. 1997). Some examples of disease reduction by primary prevention include a reduction of bladder cancers among dye workers after elimination of exposure to aromatic amines (Tomatis et al. 1990); a diminution in nasal cancers among furniture workers first employed after 1940, when exposure to wood dust was reduced (Hayes et al. 1986); and a stabilization of the incidence of pleural mesothelioma in Sweden in the 1990s, after Sweden became one of the first countries to restrict exposure to asbestos in the mid-1970s (Hemminki and Hussain 2008). Primary prevention that controls a common source of exposure to proven and probable carcinogens is far more effectual, and cost effective, than persuading thousands of persons to each change their individual behaviors (Asaria et al. 2007; Doyle et al. 2006).

Cancer and other noncommunicable diseases (NCDs) such as cardiovascular disease, chronic lung disease, and diabetes have many shared risk factors. Thus, reducing exposure to environmental and occupational carcinogens can produce important co-benefits for health. For instance, a reduction in acute coronary events has been observed after the institution of smoke-free policies in public places (Cesaroni et al. 2008). Control measures to reduce outdoor air pollution from motor vehicle traffic decrease exposure to diesel exhaust gases and contribute to a reduction in cardiovascular and nonmalignant respiratory morbidity as well as a reduction of lung cancer. Banning the use of asbestos will prevent cases of lung cancer and mesothelioma (Hemminki and Hussain 2008) as well as asbestosis, a nonmalignant fibrotic condition of the lungs. Improved urban traffic policies often reduce traffic accidents and injuries; they may also lead to the promotion of physical exercise, which is protective against a number of cancers (WHO 2006a). Environmental and occupational policies that prevent cancer also have social and economic benefits. The implementation by the U.S. Environmental Protection Agency (EPA) of national air quality control measures mandated by the Clean Air Act (initially in 1970, and strengthened in 1977 and 1990) (Clean Air Act 1970) generated substantial economic, environmental, and health benefits: air pollution was reduced, decreasing the burden of cancer and other diseases (U.S. EPA 2011). California is currently setting out the Safe Consumer Products regulations, one example of a U.S. regulation initiative at the subnational level on safer use of chemical products, which is a further step designed to counter chemical exposure–related diseases such as cancer (Brown 2012).

Primary prevention offers the most cost-effective approach to reducing cancer and other NCDs; however, primary prevention has been often neglected while secondary prevention and treatment have been given priority, partly because the results of primary prevention are difficult to recognize in individuals and because its impact may take several decades to emerge (Adami et al. 2001). In 2012, the new cases of cancer were estimated globally to cost US$ 154 billion in medical expenses (53% of the total costs) (Bloom et al. 2011). NCDs pose a substantial human and economic burden worldwide. It is estimated that NCDs will cost US$ 47 trillion over the next 20 years (Bloom et al. 2011), nevertheless, cancer and other NCD prevention has been a low priority for development agencies, governments, and other organizations (Beaglehole et al. 2011). In June 2012, the outcome document of the Rio+20 Conference on Sustainable Development acknowledged that “the global burden and threat of NCDs constitutes one of the major challenges for sustainable development in the 21st century” and “health is a precondition for, an outcome of, and an indicator of all three dimensions [economic, social, and environmental] of sustainable development” (United Nations 2012). Arguably, governments should make a strategic focus for development and sustainability by securing and promoting the health and well-being of current generations without compromising the ability of future generations to meet their own needs (World Commission on Environment and Development 1987).

The main objective of this review was to present an evidence-based global strategy for the primary prevention of environmental and occupational cancer. Here we highlight the need for, and the feasibility of, a common global vision for primary prevention.

## Methods

We developed this strategy by systematically reviewing policy approaches and effective interventions currently available for the primary prevention of cancer. Relevant studies from January 1980 through October 2012 were identified through PubMed by use of combinations of the search terms “environmental,” “occupational,” “exposure,” “cancer,” “primary prevention,” and “interventions.” We also searched the reference lists of selected articles (e.g., reviews) and reports from governmental institutions and nongovernmental organizations. In addition, we took account of consultation internationally by WHO with scientists and public health experts. To supplement the literature review and to stimulate action to tackle known and preventable causes of cancer, we convened an international conference titled “Environmental and Occupational Determinants of Cancer. Interventions for Primary Prevention,” organized by the WHO in Asturias, Spain, on 17–18 March 2011 (WHO 2011a). The objective of the conference was to introduce mitigation of environmental and occupational exposures into the global agenda for preventing cancer and NCDs. The goal of the conference was to identify actions, particularly from non-health sectors, that could contribute to the inclusion of primary prevention of environmental and occupational cancer in all policies.

## Results and Discussion

*Existing policies and interventions to be enforced.* Environmental and occupational policy approaches benefit large numbers of people exposed to environmental and work hazards, and they complement individual-level programs. People may be exposed to hazardous agents in their homes, at their workplaces, in schools, and in health-care and recreational settings and, in many cases, without related acute symptoms or the possibility of identification of the involved hazard. One example of such exposure is diesel engine exhaust from vehicles or power generators, which has recently been classified as Group 1 (carcinogenic to humans) by IARC (2012); furthermore, IARC suggested regulatory measures to reduce exposure. Another example is chemicals such as colorants used widely in beverages or plasticizers; these materials have shown carcinogenicity in animal tests (Grosse et al. 2011). Persistent organic pollutants (POPs) provide a third example. Exposure of large segments of the general population to POPs occurs daily throughout life, generally at low doses, and mostly through the fat components of diet (National Research Council 2003; Patandin et al. 1999; Porta et al. 2008; United Nations Environment Programme 2003). Numerous studies have documented the presence of POP residues in many types of foods (Bocio and Domingo 2005; Darnerud et al. 2006; Fattore et al. 2008; National Research Council 2003; Patandin et al. 1999; Schafer and Kegley 2002; Schaum et al. 2003; Schecter et al. 2010). In circumstances of widespread and mostly “invisible” exposure such as these, only cross-sectorial policies, namely policies that work across different sectors (i.e., from health, food, and environmental to housing, energy, and industrial policies) can be effective at controlling chemical contamination of human and animal food chains.

Occupational exposures to carcinogens—including formaldehyde; solvents such as benzene; metals such as arsenic, cadmium, and chromium IV; and mineral oils—are avoidable risks. Workers are generally exposed involuntarily to these occupational carcinogens. Although occupationally related cancer represents only a modest portion of the total number of cancer cases on a global scale, it may in fact cause a substantial proportion of cancer cases among certain groups of workers. The lifelong contribution to the occurrence of cancer (and other disorders of complex etiology) of exposure to epigenetic and indirectly genotoxic agents in the workplace and elsewhere is receiving increasing attention (Barouki et al. 2012; Henkler and Luch 2011; Hernández et al. 2009; Hou et al. 2012; Jirtle and Skinner 2007; Lee et al. 2009; Manikkam et al. 2012; Soto and Sonnenschein 2010; Vandenberg et al. 2012). Primary prevention of occupational cancer requires explicit social security, labor, and health legislation. While great achievements in occupational safety and hygiene have been made in some parts of the world, there is less worker protection in others, particularly in countries where workers have little choice and scant social and/or political influence (Loewenson 2001; Mamuya et al. 2006; McCormack and Schüz 2011).

*Generic principles.* Primary prevention strategies need to be prioritized today because their full benefit will only be effective in the future, often decades after their introduction, due to the long latency periods in the development of cancer. This can be illustrated with the example of the asbestos ban in the United Kingdom; even with a ban and removal from buildings starting in 1999, the peak in mesothelioma occurrence is predicted to happen no sooner than 2016 (Tan et al. 2010). In situations lacking definitive scientific evidence of causality but with the suspicion of a link with an increased risk of cancer, some generic principles may assist policy makers facing public health and environment decisions. The application of the precautionary principle of “where there are threats of serious or irreversible damage, lack of full scientific certainty shall not be used as a reason for postponing cost-effective measures to prevent environmental degradation” (United Nations Conference on the Environment and Development 1992) and of the “ALARA (as low as reasonably achievable) principle for exposures, are examples of such approaches. Disseminating information and advocacy materials to raise awareness about environmental risks and work hazards are also worthwhile strategies.

*Existing policies and legislative tools to prevent environmental and occupational risks related to cancer.* Reviewing the existing scientific literature and policy approaches and interventions for the primary prevention of cancer, we found that a rich body of legislation, regulations, and policies for eliminating or reducing exposure to carcinogens exists at both national and international levels. Examples related to chemical exposures are summarized in the Appendix (European Commission 2012; Massachusetts Department of Environmental Protection 1989; National Committee on Environmental and Occupational Exposures 2006; Nudelman et al. 2009; President’s Cancer Panel 2010; United Nations Economic Commission for Europe 2003). Examples of specific bans of chemicals include prohibitions on the use or export of asbestos (using economic and technological mechanisms to encourage replacing asbestos with available safer substitutes); the cessation of arsenic pesticides use and the banning of cosmetic pesticides use in residential lawns and gardens (President’s Cancer Panel 2010); and the banning of smoking in indoor workplaces, public transport, and indoor public places (WHO 2009c). Examples of transectorial economic policies resulting in health benefits include promoting the use of clean burning and efficient stoves, improving stoves where access to alternative fuels is limited, and improving ventilation, kitchen design, and stove placement to avoid exposure to indoor smoke (Lan et al. 2002) as well as expanding public and alternative transportation systems, improving urban planning to reduce the need for motorized transport, and adding more pedestrian-oriented streets to reduce traffic-related air pollution (WHO 2006b).

Regarding radiation exposures, several measures or proposals oriented to avoid ionizing radiation exposures in occupational and medical settings are listed in the Appendix (National Committee on Environmental and Occupational Exposures 2006; Nudelman et al. 2009; President’s Cancer Panel 2010).

In the case of radon, WHO (2009b) recommendations include increasing ventilation in enclosed spaces where radon accumulates, reducing negative pressures within buildings to prevent inflow of radon from the ground, and setting national radon programs. These programs may include noteworthy measures such as establishing national reference levels, identifying geographical areas, effective risk communication, collaborating with other health promotion programs (e.g., indoor air quality, tobacco control), ensuring professional competence in prevention and mitigation of radon exposure, establishing building codes (e.g., installation of preventive measures in homes under construction, radon measurement during purchase and sale) (WHO 2009b).

On the other hand, increasing the provision of shade in public areas and other measures to reduce ultraviolet (UV) exposure, and banning unsupervised tanning beds, and prohibiting tanning bed access for minors are measures already in place in several countries (Makin and Dobbinson 2009; Mitchell 2010; Nordqvist 2008; Teich 2010; Vaidyanathan 2009).

Fifteen industrial processes or occupations such as the rubber industry, iron and steel founding, and painting have been classified by the IARC as falling within Group 1 (carcinogenic to humans) (IARC 2008). Occupational cancer directly caused by or related to recognized carcinogens tends to be concentrated among relatively small groups of persons, among whom the individual risk of developing the disease may be quite high. These cancers are almost entirely preventable by eliminating or reducing the relevant exposure, substituting safer materials for carcinogenic materials, or in some cases, adjusting industrial processes and ventilation or providing worker protection to avoid direct contact with the carcinogen. Measures to control work hazards should therefore have a high priority in any program of cancer prevention, even if they are responsible for only a small proportion of all cancers. Measures may include those described by the National Committee on Environmental and Occupational Exposures (2006), Nudelman et al. (2009), O’Neill (2007), the President’s Cancer Panel (2010), and WHO (2006a, 2009c). [For a list of example control measures, see Supplemental Material (pp. 2–3 (http://dx.doi.org/10.1289/ehp.1205897).] A useful strategy for each jurisdiction is to assess systematically the range and hierarchy of cancer risks to which individuals are exposed. Subsequently, a systematic process can be established to act first on the carcinogens with the highest risk and widest reach, and then work progressively through the prioritized list. Policy makers in a number of countries are working intensively to develop public policies and cancer prevention programs to create occupational exposure matrixes (OEMs) and information systems on cancer exposures such as CAREX (CARcinogen EXposure), for which Finland was the pioneer (Finnish Institute of Occupational Health 2010) and which other countries such as Canada, Costa Rica, and the countries of the European Union have now adopted (Health Canada 2011; Kauppinen et al. 2000; Partanen et al. 2003).

*Environmental measures to help individuals protect themselves can work together with population-based public policies.* Environmental and occupational interventions for primary prevention of cancer and other NCDs must also be directed at individuals. Many members of the public remain unaware of common environmental carcinogens such as radon and even secondhand smoke or manufacturing and combustion by-products that are released into the environment. Environmental and occupational risk communication should be emphasized; public awareness and perception of risk can be improved using social marketing techniques and by involving the media. For example, school-based programs focused on preventing skin cancer could target vulnerable populations, such as children and fair-skinned individuals, and encourage them to avoid too much sunlight at midday and to use personal protection measures. An example of improving individual and community behaviors concerning sun protection was the Pool Cool program (Glanz et al. 2002) in the United States, an educational prevention program against skin cancer directed at children enrolled in swim lessons, their parents, and staff at outdoor swimming pools. Reasons for successful implementation included the provision of a toolkit, ease of implementing measures, and field coordinators’ support. As social norms, policies, and participation in the program increased, sunburns tended to decrease; protective behaviors have also been effective among outdoor workers (Escoffery et al. 2008, 2009; Hall et al. 2009). Another example is the SunSmart Schools program in Australia (Jones et al. 2008).

Medical procedures involving exposure to ionizing radiation have both risks and benefits. Although the benefits normally outweigh the risks, patients are entitled to be informed and physicians are advised to minimize unnecessary exposure. Recently, a relationship between computer tomography and childhood cancer risk has been observed (Pearce et al. 2012) that could be reduced by appropriate dose optimization for children. These issues deserve an even more sensitive approach when they affect secondary prevention interventions (e.g., mammography for early detection of asymptomatic breast cancer) (Nudelman et al. 2009; President’s Cancer Panel 2010; WHO 2006a). Another example is advising the public about the benefits of different radon prevention and remedial actions to control radon in dwellings (such as checking levels of radon, installing a ventilation system in the basement) (WHO 2009b). Informing the public about the benefits of reducing exposures to pollutants for the prevention of cancer and other NCDs will empower civil society to request action on issues that are, for the most part, out of an individual’s control (e.g., urban air pollution, smoking in public places, increasing shade in public places in high UV radiation climates). Institutions and organizations can also facilitate individual behaviors that decrease cancer incidence, for example, encouraging consumers to reduce household use of hazardous chemicals; to use public and ecological transportation; to ventilate rooms or work outside when using solvents; and to minimize contact with pesticides during gardening and outdoor activities. Furthermore, public health advocacy by citizens’ groups could help change corporate practices. Public disclosure of corporations that utilize or permit human exposure to carcinogens could contribute to more responsible consumer behaviors and corporate practices.

Finally, it has been demonstrated that public policies such as legislation on smokefree workplaces not only protect nonsmokers from the dangers of secondhand smoke, but they also create an environment that encourages smokers to reduce or stop smoking (Fichtenberg and Glantz 2002). It needs to be noted however that active pressure should only be encouraged for established carcinogens with the guidance of public health specialists, as the public perception of risks does not always correspond to the true harmfulness of an agent, as for instance in the case of electromagnetic fields. Table 1 presents nine risk factors for occupational and environmental-related cancers and our perception of the state of the evidence concerning measures that support primary prevention, and highlighting key areas that need to be strengthened.

**Table 1:** Summary of nine environmental and occupational risk factors for cancer: areas to be strengthened.

Risk	Scientific evidence in support of causationa	Awareness-raising measuresb	Existence of policies/recommendationsc	Existence of legislationd	Level of advocacy for primary preventione	Implementation of policies and legislationf	Public perception of riskg
Asbestos	High	High	High	High	High	Intermediate	Intermediate
POPs	Intermediate	Low	High	Intermediate	Intermediate	High	Low
Indoor radon	High	Intermediate	High	Intermediate	Intermediate	Intermediate	Low
Outdoor air pollution/diesel exhaust	High	High	High	Intermediate	Intermediate	Intermediate	Intermediate
Indoor emissions from household combustion	Intermediate	High	High	Intermediate	Low	Intermediate	Low
Secondhand smoke	High	High	High	Intermediate	Intermediate	Intermediate	Intermediate
Ionizing radiation (medical exposure)	High	Low	Intermediate	Low	Low	Intermediate	Low
UV and tanning beds	High	High	High	Intermediate	Intermediate	Intermediate	Intermediate
Electromagnetic fields	Low	Intermediate	Low	Low	Low	Low	High
POPs, persistent organic pollutants. The methodology followed to classify the risk factors combined a review of relevant literature, consultation with scientists and public health experts, and consensus reached among participants in the WHO International Conference on “Environmental and Occupational Determinants of Cancer. Interventions for Primary Prevention” (17–18 March 2011, Asturias, Spain) (WHO 2011a). aAmount of scientific evidence in support of causation. bNumber of awareness-raising measures (e.g., campaigns) at national and/or international level. cExtent of governmental or nongovernmental policies, understood as principles or rules, and/or recommendations at the national and/or international level. dExistence of legislation at national and/or international level. eLevel of advocacy (governmental and nongovernmental) for primary prevention of cancer at national and/or international level. fLevel of implementation of policies and/or legislation at national and/or international level. gLevel of the perception of risk held by the general population versus the actual amount of scientific evidence in support of causation.							

*From integration of environmental and occupational causes of cancer into the global cancer agenda to broadening to cancer prevention in all policies.* The conference held in Asturias, Spain, on 17–18 March 2011 reinforced the understanding that many cancers of environmental and occupational origin such as lung cancer, mesothelioma, and melanoma are preventable and advocated for integrating primary prevention of environmental and occupational cancers into a global cancer agenda. The conference recommended that more emphasis be placed on including rigorous primary prevention strategies in cancer control policies. Because cancer is a global public health problem, prevention should be part of all policies: That is, the potential effects of any policy, particularly regarding the development of cancer, should be considered before its implementation by policy makers. Growing awareness about environmental and occupational risk factors for cancer has led policy makers in many countries to take actions for primary prevention. For example, bans and restrictions on the production, marketing, and use of some major carcinogens, such as asbestos and secondhand smoke from tobacco, have been implemented. However, an unacceptable consequence of measures taken at national or regional levels (e.g., by the European Union) has been the transfer of carcinogenic materials to countries lacking effective cancer prevention policies. Companies based in developed countries often employ less stringent controls on carcinogens in their factories located in developing countries if not otherwise forced by national regulation (Castleman 1980; Castleman et al. 2008; Jeyaratnam 1994; Park et al. 2009). Thus, international efforts are required to reduce global cancer rates.

On the other hand, promoting research has provided ample evidence that supports effective prevention strategies to decrease the global incidence and prevalence of cancer (Hiatt and Rimer 1999). However, a large number of environmental exposures are understudied and therefore remain classified as being possibly carcinogenic. Knowledge is also limited on the consequences of cumulative lifetime exposure to carcinogens, relevant time windows of exposure (e.g., early life), and on the interaction of multiple concurrent exposures (Nudelman et al. 2009; President’s Cancer Panel 2010). In addition, further research is needed on the impact of environmental and occupational exposures in low- and medium-income countries, which often have higher exposure levels or higher lifetime cumulative exposure, lesser protection levels, or different exposure patterns (e.g., an earlier age at first exposure because of child labor) compared to high-income countries that currently provide most of the data (McCormack and Schüz 2011). Moreover, for some cancers there is little knowledge on their etiology, and further research is needed to disentangle the role of the environment in their causation. There is emerging evidence that societal efforts to decrease exposure to carcinogens have positive impacts on quality of life, productivity, economic growth, social cohesion, and environmental capital (Oberg et al. 2011; U.S. EPA 2011; Venkataraman et al. 2010). The cancer prevention agenda must be broadened to include research on these issues by social and political sciences. Implementation science deserves particular attention in order to ensure that the knowledge generated is integrated effectively into decisions and policies that affect cancer and that the delivery of cancer prevention policies reaches vulnerable communities, especially in the developing world (Madon et al. 2007). Influence and advocacy for primary prevention of cancer should also be underpinned by research (Brownson et al. 2011).

Finally, establishing linkages between public health programs for the prevention of cancer and programs in occupational health, environmental health, chemical safety, and food safety will create synergies and, as a result, assist governments, industry, workers and their organizations, the health-care sector, nongovernmental organizations, advocacy groups—and individuals themselves—to achieve benefits in a range of areas (such as manufacturing, energy and mining, transportation, and housing). Linkages of this nature can be envisaged in the context of cross-sectorial initiatives or strategies such as “Health in All Policies” (Ståhl et al. 2006). It would seem good sense to put cancer prevention “in all policies.”

*Policy framework: gaps and opportunities.* Historically, there has typically been a delay between the establishment of scientific evidence and action taken to reduce exposure to environmental and occupational risks. Only a limited amount of research has been translated into primary prevention policies. Even substances whose dangers are thoroughly documented, such as asbestos, are still used in many countries (European Environment Agency 2001). In other situations, there is still a lack of compelling evidence and therefore further research is needed. Some priority areas are listed in Supplemental Material, pp. 4–6 (http://dx.doi.org/10.1289/ehp.1205897).

In order to design an appropriate roadmap for primary prevention of environmental cancer, measures taken in some areas need to be strengthened. Table 1 summarizes the state at which nine environmental and occupational risks stand in a public health roadmap for primary prevention of cancer. The table reflects our views after reviewing the relevant literature and consulting with scientists and public health experts.

Identifying efficient means to implement existing environmental and occupational interventions is crucial for the development of a policy framework for primary prevention. Based on the recommendations reached at the international conference, “Environmental and Occupational Determinants of Cancer: Interventions for Primary Prevention,” we have outlined some of the components that the proposed framework could include:

The development or adaptation of appropriate tools for screening to identify the main risks for cancer and other NCDs in specific communities or sectors. This implies the identification of settings such as households, hospitals, industries; the use of available methodologies and techniques (e.g., a control banding tool for hazardous chemicals) and the definition of actions linked to interventions or mitigation measures to reduce environmental and occupational exposures.
Building capacity of health-care workers, construction experts, occupational hygienists, and others who have to use the tools in those settings or sectors.
The use of screening tools for specific situations (e.g., using a health impact assessment in planning activities, evaluating existing interventions and activities to determine what can be modified and improved).
Monitoring and evaluating progress in the implementation of primary prevention activities.
Reporting to the setting or sector on progress made.

## Conclusions

Cancer is a major problem worldwide. It causes severe and long-term human suffering for individuals and families. It has enormous economic impacts on society. It creates high costs for health-care systems and, in fact, causes the highest economic loss of all the 15 leading causes of death worldwide. The global economic impact of premature death and disability from cancer in 2008 was US$ 895 billion, not including direct costs of treatment (John and Ross 2010).

A substantial proportion of all cancers is attributable to carcinogenic exposures in the environment and the workplace, and is influenced by activities in all economic and social sectors. Many of these exposures are involuntary but can be controlled or eliminated through enacting and enforcing proactive strategies for primary prevention.

The primary prevention of cancers of environmental and occupational origin reduces cancer incidence and mortality and is highly cost effective; in fact, it is not just socially beneficial because it reduces medical and other costs, but because it averts the suffering of many human beings. It requires establishing a multisectorial approach and multiple partnerships. Commitment is essential from health and non-health sectors (such as the environment, labor, housing, transport, industry, and trade sectors), community organizations, private enterprises, health and workers’ compensation and insurance organizations, and other key actors at the national and international levels. All stakeholders should be involved in developing strategies to combat the environmental and occupational causes of cancer and to secure commitment to policy change at governmental levels.

Currently, in most countries the almost exclusive focus of cancer policies is on secondary prevention (i.e., early detection), diagnosis, and treatment. As shown in Table 1 regarding the existence and implementation of legislation or the level of advocacy, insufficient resources are devoted to primary prevention, which aims to eliminate or control exposures to environmental and occupational carcinogens. The prevailing approach is socially unfair and often unsustainable, especially in low- and middle-income countries. Opportunities should be taken to focus the global policy agenda for cancer and other NCDs in the direction of primary prevention through environmental and occupational interventions. It is crucial therefore to *a*) lay the political foundations by raising awareness that cancer control is not only about treatment, and *b*) identify innovative ways to invest in prevention through cross-sectorial collaboration.

There is sufficient evidence that primary prevention is feasible and highly effective in reducing cancer incidence. To create a blueprint for the inclusion of strategies for primary prevention of cancer of environmental and occupational origin in national cancer policies in countries around the world, we organized the WHO international conference where the “Asturias Declaration: A Call to Action” was developed (WHO 2011a). The declaration aims to introduce the mitigation of environmental and occupational exposures into the global agenda for cancer and other NCDs. The declaration of Asturias states that

Actions for primary prevention of cancer of environmental and occupational origin are still uncoordinated and do not make full use of existing knowledge about primary prevention.

There is a need to create a global strategic framework for control of environmental and occupational carcinogens that enables and promotes primary prevention more broadly.

Global strategic framework should make use of existing tools and knowledge, and would require *a*) developing and implementing screening tools to identify the main risks of cancer and other NCDs in specific settings; *b*) capacity building of the actors involved in implementation; *c*) using existing opportunities such as legislation and regulations that need to be adopted and enforced by all countries to protect their populations; *d*) tailoring risk communication about primary prevention to local circumstances and educating populations about the respective prevention strategies available; and *e*) monitoring, evaluating, and reporting on the progress made.

This review, set in the context of the consensus reached at the First Global Ministerial Conference “Healthy Lifestyles and Noncommunicable Disease Control” (WHO 2011b), held in Moscow in April 2011; at the United Nations General Assembly High-level Meeting “Prevention and Control of Noncommunicable Diseases” (United Nations 2011), held in New York in September 2011; and at the WHO Executive Board meeting (WHO 2012), held in Geneva in January 2012, provides a firm basis on which to put forward primary prevention as a substantive strategic approach for the sustainable development agenda of governments and to include it as part of a framework of action in both health and non-health policies.

## Appendix

Examples of regulations and policies related to chemical exposures

1. General measures to avoid chemical exposures
2. Regulations for substitution and phasing out of replaceable processes or carcinogenic substances in the workplace, by replacing them with less dangerous substances
3. Measures aimed at closing industrial facilities in which carcinogens are released, wet processes, ventilation, filtration or cleaning
4. Controlling carcinogen exposure based on threshold limit values
5. Offering incentives to corporations to encourage the elimination of harmful chemicals in their products and processes
6. Disclosure-labeling laws for identification and classification of chemicals by types of hazard, including safety data sheets
7. Setting accreditation procedures for labeling industries as health sensible, and encouraging public administrations to establish preferential contracts with those companies
8. Promoting effective measures to ensure the safe storage and disposal or recycling of chemicals
9. Regulations ensuring the safe management of hazardous substances during trade and transport.

Examples of measures or proposals oriented to avoid ionizing radiation exposures in occupational and medical settings

1. The harmonization of standards for radiation protection {e.g., International Basic Safety Standards for Protection against Ionizing Radiation and for the Safety of Radiation Sources [co-sponsored by the International Atomic Energy Agency (IAEA), WHO, Pan American Health Organizatio (PAHO), International Labour Organization (ILO), Food and Agriculture Organization (FAO), and Nuclear Energy Agency/Organization for Economic Co-operation and Development (NEA/OECD)] [IAEA 1996]}
2. The design of public policies, including legislation, to promote appropriate justification of radiological medical procedures to avoid unnecessary exposures
3. Education of physicians to promote the use of referral guidelines as decision-making tools to justify diagnostic procedures of choice
4. Education and training of imaging professionals (radiologists, nuclear medicine physicians, medical physicists and technicians) to apply diagnostic reference levels to radiological procedures, to reduce radiation doses without affecting image quality
5. Regulations for occupational radiation protection (e.g., shielding, time and distance to the source, limits for the effective dose in workers of 20 mSv/y) and dose-monitoring systems

## Supplemental Material

(176 KB) PDF
