# Supplementary material for: Environmental and Occupational Interventions for Primary Prevention of Cancer: A Cross-Sectorial Policy Framework
Source: Environ Health Perspect. 2013 Feb 5;121(4):420–6. doi: 10.1289/ehp.1205897 (PMC3620754; doi:10.1289/ehp.1205897)
Supplement: (176 KB) PDF [file ehp.1205897.s001.pdf]

Environmental Health Perspectives doi:10.1289/ehp.1205897

SUPPLEMENTAL MATERIAL

Environmental and Occupational Interventions for Primary Prevention of Cancer: a Cross-Sectorial Policy Framework

Carolina Espina, Miquel Porta, Joachim Schüz, Ildefonso Hernández Aguado, Robert V. Percival, Carlos Dora, Terry Slevin, Julietta Rodriguez Guzman, Tim Meredith, Philip J. Landrigan, Maria Neira.

Table of contents:

Supplemental Material: Examples of control measures in the work environment 2

General measures: 2

Measures for chemical exposures: 2

Measures for physical exposures: 3

Supplemental Material: Examples of gaps in knowledge and research, and implementation of environmental and occupational interventions. 4

In knowledge and research: 4

In implementation: 5

In the work environment: 6

Some specific examples: 6

REFERENCES 7

Supplemental Material: Examples of control measures in the work environment

General measures:

- Hazard identification, risk assessment, risk management and risk communication in all occupational settings;
- Identification and surveillance of exposure, and health surveillance: estimating the number of workers who come into contact with substances and are employed in occupations and industries with increased carcinogenic risk, developing a prioritized strategy aimed at systematically reducing to a minimum the number of workers exposed, and the duration and degree of exposure, and establishing an appropriate system of records;
- Regulating the use of protective equipment for workers and decontamination facilities in industries;
- Raising the overall awareness of workers, employers, health and safety professionals about work hazards, their recognition and elimination, and ensuring the reporting of cancer cases with a suspected occupational etiology;
- Raising awareness among health professionals (clinicians and public health officers) about the links between environmental and occupational exposures, and cancer disease;
- Inclusion of occupational cancer in national lists of occupational diseases and ensuring that physicians report all such cases;
- Integration of prevention of occupational cancer with overall cancer control programs.

Measures for chemical exposures:

- Establishing recommendations for proper labeling, use and handling of chemicals (in industries, agriculture and also in trade schools), and training packages for workers for technical prevention and personal hygiene;

- Empowering workers by providing them with access to information about their exposures and risks (e.g. increasing access to the International Chemical Safety Cards), and assuring compliance with their right to know about their exposures to carcinogens at work.

Measures for physical exposures:

- Implementing regular radiation safety training programs for workers;
- Providing monitoring services at the workplace and assessing occupational radiation exposures;
- Establishing national dose registries for radiation and information systems on occupational exposures;
- Promoting radiation safety culture and raising awareness about radiation among health-care workers (e.g. interventional radiologists and nuclear medicine professionals).

Supplemental Material: Examples of gaps in knowledge and research, and implementation of environmental and occupational interventions.

In knowledge and research:

- More research on cancers with few established risk factors, ideally in high incidence areas; continue research into possible but not established carcinogens ideally in areas of high exposure levels;
- Studying the interplay between timing of exposure and multiple exposures, and mixtures;
- Studying low-level exposures to carcinogenic pollutants because of the multiplicity of substances, the involuntary nature of many exposures, and the potential that even low-level exposures may contribute to the cancer burden when large numbers of people are exposed;
- Studying genetic susceptibility to environmental risks;
- Identifying carcinogenic exposures in work places not under surveillance for possible hazards;
- Identifying special populations at risk and their cancer burden, e.g. children;
- Lack of reliable epidemiological data on dose-response relationships, and therefore problems with setting protection guidelines;
- Further research is needed to reduce uncertainties about radiation-induced cancer risks including dose response for different types of cancers, influence of gender and age, risk of prenatal exposures, interaction of radiation with chemical carcinogens, and radiation quality effects;
- Non-ionizing radiation as from mobile phones (radiofrequency) or power lines (extremely low-frequency); although little evidence for carcinogenicity, the possible effects of long-term heavy use of mobile phones requires further investigation due to widespread use in population (IARC 2011, INTERPHONE Study Group 2010);

- Further research on vitamin D in relation to UV exposure is needed;
- Further studies of the health effects of manufactured chemicals not yet fully assessed;
- Research on the politics of cancer prevention policies: barriers and opportunities at high political level to prevent cancer;
- Development of an international catalogue and tool to enact laws to prevent cancer;
- Research on environmental justice, regarding exposures to environmental and occupational carcinogens, at a global scale.

In implementation:

- Despite our increased body of scientific knowledge about cancer epidemiology, causation, and preventive measures, this knowledge has not been translated adequately into public policy initiatives that will impact public health;
- Nationwide assessment of environmental and occupational cancer risk factors is not performed systematically in all countries;
- Differences in resources and cultural contexts in low- and middle-income countries may affect the feasibility or efficacy of interventions implemented in and adapted to high-income countries;
- Priority interventions for primary prevention of cancer may differ on a smaller regional level than country-wide and need to be tailored to the local cancer burden;
- Risk communication strategies need to be targeted at policy-makers to rise their awareness that most environmental and occupational risk factors are beyond the personal control of individuals, while increasing individuals' risk perception and concern about changes in behavior so as to avoid exposure to certain substances;

- Mechanisms to measure the level of exposure to carcinogens of the general public (typically lower than those experienced in occupational settings) are often neglected;
- Lack of stringent mechanisms to reduce exposure to substances that are used under less controlled conditions (e.g. diesel motor exhaust);
- Many substances do not qualify for classification as a carcinogen by IARC because of a lack of information; carcinogen testing data should be available for many industrial and commercial chemicals and such testing should ideally be performed before products are introduced into the market;
- Primary prevention of cancer through environmental and occupational interventions is too often omitted from National Cancer Plans.

In the work environment:

- In many countries, the importance of work hazards has not been fully assessed by the corresponding occupational health institutions, and prevention of occupational cancer has not been given high priority;
- Occupational hygiene conditions in small enterprises in some high-resource countries, and in industries in low- and middle-income countries (e.g. asbestos, crystalline silica and pesticide industries) remain highly substandard.

Some specific examples:

- Using and importing asbestos is still a common practice in many (mostly developing) countries;
- Exporting asbestos still remains active in several producing countries;
- Only a few countries, such as Scotland, Belgium, Germany, Spain, France, Brazil, Canada and Australia have regulations about the use of tanning beds.

REFERENCES

International Agency for Cancer Research (IARC). 2011. IARC classifies radiofrequency electromagnetic fields as possibly carcinogenic to humans. Press release Nº 208. Lyon, France. Available: http://www.iarc.fr/en/media-centre/pr/2011/pdfs/pr208_E.pdf [accessed 21 October 2012].

INTERPHONE Study Group. 2010. Brain tumour risk in relation to mobile telephone use: results of the INTERPHONE international case-control study. Int J Epidemiol 39(3):675-694.
